# Cognitive Behavioral Therapy for Youth With Childhood‐Onset Lupus: A Randomized Clinical Trial

**DOI:** 10.1002/acr.70010

**Published:** 2026-03-20

**Authors:** Natoshia R. Cunningham, Thea Senger‐Carpenter, Jocelyn Zuckerman, Michelle Adler, Mallet R. Reid, Ashley Danguecan, Luana Flores Pereira, Sarah I. Mossad, Samantha L. Ely, Khalid Abulaban, Elizabeth A. Kessler, Natalie Rosenwasser, Tamar B. Rubinstein, Ekemini A. Ogbu, Emily A. Smitherman, Alaina Miller, Taylor Abounader, Elizabeth Ross, Livie Timmerman, Dhriti Sharma, Jennifer N. Stinson, Stacy Allen, Kabita Nanda, Tala El Tal, Deborah M. Levy, Linda T. Hiraki, Hermine I. Brunner, Mathew Reeves, Steven J. Pierce, Andrea Knight

**Affiliations:** ^1^ Michigan State University College of Human Medicine East Lansing; ^2^ The Hospital for Sick Children Toronto Ontario Canada; ^3^ Wayne State University School of Medicine Detroit Michigan; ^4^ Helen DeVos Children's Hospital Grand Rapids Michigan; ^5^ University of Washington School of Medicine Seattle; ^6^ Seattle Children's Hospital Seattle Washington; ^7^ Children's Hospital at Montefiore Bronx New York; ^8^ Albert Einstein College of Medicine Bronx New York; ^9^ Cincinnati Children's Hospital Medical Center Cincinnati Ohio; ^10^ University of Cincinnati College of Medicine Cincinnati Ohio; ^11^ University of Alabama at Birmingham; ^12^ Children's Hospital of Alabama Birmingham; ^13^ Johns Hopkins All Children's Hospital St Petersburg Florida; ^14^ MetroHealth Medical Center Cleveland Ohio; ^15^ Mott Children's Hospital Ann Arbor Michigan; ^16^ Temerty Faculty of Medicine, University of Toronto Toronto Ontario Canada; ^17^ Michigan State University Graduate School East Lansing; ^18^ Michigan State University, Center for Statistical Training and Consulting East Lansing

## Abstract

**Objective:**

Our objective was to determine the feasibility and acceptability of the Treatment and Education Approach for Childhood‐Onset Lupus (TEACH), a six‐session cognitive behavioral intervention addressing depressive, fatigue, and pain symptoms, delivered remotely to individual youth with lupus by a trained interventionist. We expected that TEACH would be considered feasible and acceptable based on recruitment and retention rates. We also examined the effect of TEACH on youths’ depressive, fatigue, and pain symptoms compared to medical treatment as usual (TAU).

**Methods:**

A pilot two‐arm longitudinal randomized controlled clinical trial was conducted. Adolescents (12–17 years) and young adults (18–22 years) with childhood‐onset systemic lupus erythematosus and elevated depressive, fatigue, and/or pain symptoms were recruited from six pediatric rheumatology sites across the United States and Canada from August 2020 to March 2023. Participants were randomized 1:1 to TEACH and TAU or TAU alone and reported symptom data at baseline and eight weeks later.

**Results:**

Of the 200 youth approached, 97 consented to participate (48.5% recruitment). Among 64 eligible participants, 32 were randomized to TEACH and TAU and 32 to TAU alone. Retention was high (92.2%). At postassessment, the intervention group demonstrated reductions in depressive (C_emm_ 7.88, 95% confidence interval 3.20–12.60; 14%) and fatigue (C_emm_ 3.91, 95% confidence interval 0.44–7.39; 7%) symptoms but not pain (C_emm_ 0.89, 95% confidence interval −0.06 to 1.84).

**Conclusion:**

This remotely delivered cognitive behavioral intervention tailored to youth with lupus was feasible and associated with reduced depressive and fatigue symptoms compared with medical TAU. Further increasing accessibility by implementing TEACH in medical settings may improve uptake and patient outcomes.

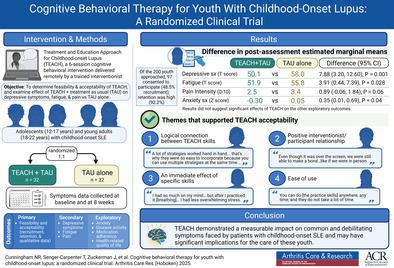

## Graphical abstract

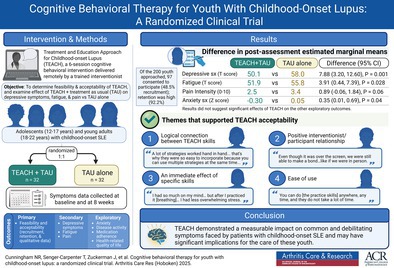

## INTRODUCTION

Childhood‐onset systemic lupus erythematosus (cSLE) is a chronic, multisystemic, autoimmune disease diagnosed before 18 years of age.^1^ Those with cSLE are at an increased risk for depressive, fatigue, and pain symptoms,^1^, ^2^ which are associated with lower health‐related quality of life (HRQoL) even when disease activity is well controlled.^3^ Female youth, as well as Asian, Native American, and Black youth, are disproportionately impacted by cSLE.^4^ Indeed, cSLE is the fifth leading cause of death for Black and Hispanic female youth aged 15 to 24 years old.^5^ Notably, Black youth may be less likely to receive effective management for comorbid symptoms (eg, anxiety, depression) compared to White peers.^6^

**SIGNIFICANCE AND INNOVATION**

- Depressive, fatigue, and pain symptoms are common among youth with childhood‐onset systemic lupus erythematosus (cSLE) and negatively impact quality of life yet remain underaddressed in current approaches to care.
- This multisite, multinational randomized control trial examined the feasibility and efficacy of the Treatment and Education Approach for Childhood‐Onset Lupus (TEACH), an individual, remotely delivered six weekly session program addressing depressive, fatigue, and pain symptoms among youth with cSLE.
- Remotely delivered TEACH is feasible and acceptable for youth with cSLE and was associated with reduced depressive and fatigue symptoms compared to treatment as usual.
- Increasing care accessibility via remote delivery may be important to depressive, fatigue, and pain outcomes among youth with cSLE.

Cognitive behavioral therapy (CBT) is effective for managing depression,^7^ fatigue,^8^ and pain^9^ in youth with other chronic medical conditions (eg, inflammatory bowel disease,^10^ juvenile fibromyalgia)^11^ and adults with SLE.^12^ To date, however, only two studies have focused on youth with cSLE. The first examined the efficacy of CBT for pain, affect, and quality of life in patients with cSLE but failed to identify clinically significant effects.^13^ The second was our previously reported, single‐arm, pilot study of the Treatment and Education Approach for Childhood‐Onset Lupus (TEACH) program.^14^ TEACH is a tailored six‐session program targeting depressive, fatigue, and pain symptoms that was delivered once a week, in person, to individual youth with cSLE.^14^ Although results indicated good retention (82.4%) with improvements in depressive and fatigue symptoms, qualitative feedback suggested that participants desired an option for remote versus in‐person delivery.^14^ The current study, therefore, examined the feasibility and acceptability of remotely delivered TEACH plus medical treatment as usual (TAU) for adolescents and young adults with cSLE. Based on our prior work among youth with cSLE^3^, ^14^ and other painful conditions (eg, functional abdominal pain disorders),^15^ we hypothesized that >65% of youth approached would agree to participate (recruitment), whereas >80% of recruited participants would complete a posttreatment assessment (retention). We also predicted that qualitative interviews with participants would support programmatic acceptability and sought to determine the efficacy of TEACH and TAU for depressive, fatigue, and pain symptoms compared to TAU alone. Finally, we explored differences in disease activity, medication adherence, and anxiety symptoms based on treatment allocation.

## PATIENTS AND METHODS

This is an international, multisite parallel pilot randomized clinical trial. All procedures were approved by the Michigan State University institutional review board (STUDY00003882). The study was preregistered at ClinicalTrials.gov (NCT04335643) and follows the Consolidated Standards of Reporting Trials (CONSORT) reporting guidelines.^15^

### Study design and participants

**Figure 1:**
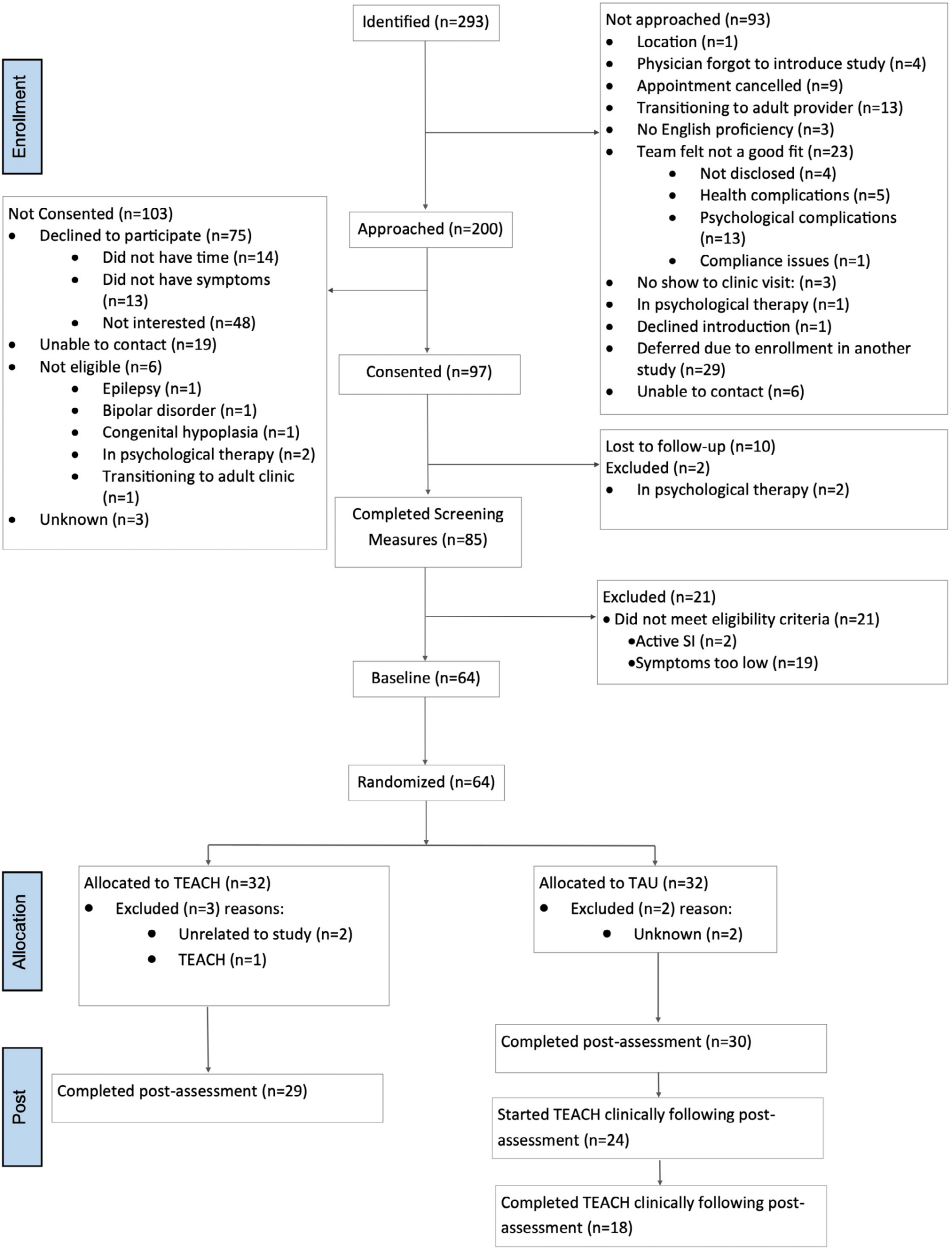
Consolidated Standards of Reporting Trials (CONSORT) diagram depicting participant flow through the trial. Of the 200 approached, 97 youth consented to participate and 85 completed screening measures. A total of 64 participants were randomized to TEACH and medical treatment (TAU) as usual (n = 32) or TAU alone (n = 32) conditions. A total of 29 youth completed TEACH and TAU and an eight‐week postassessment, whereas 30 participants with TAU completed a postassessment, 18 of whom received TEACH and TAU clinically after this. SI, sucidal ideation; TAU, treatment as usual; TEACH, Treatment and Education Approach for Childhood‐Onset Lupus.

Our recruitment and consent/assent procedures are detailed elsewhere.^16^ Briefly, youth with cSLE were recruited and consented from five outpatient pediatric rheumatology clinics in the United States and one in Canada from August 2020 to March 2023 (see Figure 1). Participants were compensated up to $100 in Amazon gift cards, with $50 distributed at baseline and $50 at the postassessment. Inclusion criteria were age between 12 and 22 years; cSLE diagnosis from a pediatric rheumatologist by age 18 years; elevated depressive, fatigue, and/or pain symptoms; and English language proficiency. Exclusion criteria included other chronic medical conditions (eg, juvenile idiopathic arthritis), documented developmental delays or severe cognitive impairments, and untreated major psychiatric illness (eg, bipolar disorder, active suicidal ideation). Youth currently engaged in psychological treatment for depressive, fatigue, or pain symptoms were also excluded to optimize detection of intervention effects.

### Intervention

The TEACH program was developed by Cunningham and colleagues^14^, ^16^ to address depressive, fatigue, and pain symptoms in youth with cSLE using cognitive behavioral and mindfulness meditation strategies.^8^, ^17^ TEACH was adapted for remote delivery with input from an advisory group of patients with cSLE and caregivers and consisted of six weekly telehealth video conferencing sessions lasting one hour each with supporting online modules and caregiver involvement for minors. Sessions were delivered by one of three trained interventionists (ie, two licensed clinical social workers or a pediatric psychologist) and comprised psychoeducation, behavioral (eg, relaxation training; activity pacing), cognitive (eg, identifying and challenging automatic thoughts; problem solving), and mindfulness strategies (see Supplementary Figure 1 as well as our published protocol paper for additional detail).^14^, ^17^ All intervention sessions were video recorded and assessed for fidelity to the TEACH manual by trained independent evaluators (eg, a physician, licensed social worker, or doctoral student of psychology). Fidelity was scored 0% to 100% based on a standardized checklist (eg, session skills and homework review; confirmation of participant understanding).

### Randomization, data collection, and blinding

Participants were randomized to receive TEACH and TAU or TAU alone on a 1:1 ratio using a stratified randomized block design, with country (United States or Canada) and age (12–17 years “adolescent,” or 18–22 years “young adult”) as strata and random blocks of four or eight. Randomization was done by a statistician who did not participate in study analyses. To maintain staff blinding to the greatest extent possible, an interventionist communicated treatment allocation directly to participants. All participants continued to receive medical TAU, and those randomized to TAU alone were offered TEACH following completion of their postassessment (eg, eight weeks after baseline). Participants (and adolescents’ caregivers) completed surveys at baseline and postassessment using REDCap.^18^ The investigators (with the exception of the interventionists and randomizing statistician) were blind to randomization throughout.

### Treatment feasibility (primary outcomes)

Feasibility was assessed using recruitment and retention rates as well as qualitative interviews, as per our prior research.^14^ Recruitment was defined as the proportion of approached youth who agreed to participate. Retention was measured by the proportion of randomized participants who completed the eight‐week postassessment. All TEACH completers (ie, those originally randomized to TEACH as well as those who completed the program clinically after the postassessment) were invited to complete a brief interview assessing programmatic feasibility and acceptability at their eight‐week postassessment. Interviews were conducted by one of two doctoral students in psychology. The interview guide, which included questions such as, “Can you integrate [these] skills into your schedule with school/work?” is based on our earlier work and is shown in Supplementary Table 1.^14^, ^19^ Participants also reported whether differences between their own and the interventionist's demographics (eg, sex, race) impacted their perception of care.

### Treatment efficacy (secondary outcomes measured at baseline and postassessment)

#### Depressive symptoms

The Children's Depression Inventory 2nd Edition (CDI‐II) and Beck Depression Inventory II (BDI‐II) assessed past two‐week depressive symptoms for adolescents and young adults, respectively.^20^, ^21^ The 28‐item CDI‐II has a possible total score range of 0 to 56 (0–2 per item), whereas the 21‐item BDI‐II ranges from 0 to 63 (0–3 per item), with higher scores indicating more severe symptoms. A 50% reduction in total score is considered clinically meaningful.^22^ Total scores were converted to T‐scores (mean ± SD, 50 ± 10) for comparability, with T‐scores ≥60 and <90 indicating study eligibility.^14^, ^16^

#### Fatigue

The Patient‐Reported Outcomes Measurement Information System Pediatric or Adult Fatigue Short Form assessed past‐week fatigue.^23^, ^24^ A total of 10 (pediatric version, completed by adolescents) or 8 (adult version, completed by young adults) items were rated on a five‐point Likert scale (never/not at all, to almost always/very much) and summed, then converted into T‐scores. T‐scores ≥60 indicated clinically elevated fatigue and study eligibility, with ≥10‐point reductions in T‐score being considered clinically significant.^25^

#### Pain intensity

Participants evaluated their average pain intensity over the past two weeks using the Pain Visual Analog Scale, which has been validated for these age groups.^26^ Values range from 0 (no pain) to 10 (worst imaginable pain), with ratings ≥3 indicating moderate pain^27^ and study eligibility. Relative reductions of ≥30% in pain intensity scores are clinically significant.^28^

### Exploratory outcomes

#### Anxiety

Participants reported their past three‐month anxiety symptoms using the 41‐item Screen for Child Anxiety Related Disorders (SCARED; completed by adolescents)^29^ or 44‐item Screen for Adult Anxiety Related Disorders (SCAARED; completed by young adults).^30^ Items are scored on a three‐point Likert scale (0 = not true or hardly ever true, 1 = somewhat or sometimes true, 2 = very true or often true) and summed to yield total scores of 0 to 82 (SCARED) or 0 to 88 (SCAARED). Total scores were transformed into Z‐scores for comparability, as recommended by the measure developer (Boris Birmaher, MD, email communication, November 6, 2023). A reduction of >50% is meaningful.^31^

#### Disease activity

Site rheumatologists recorded disease activity using the Systemic Lupus Erythematosus Disease Activity Index 2000.^32^ Scores range from 0 to 105, with scores ≥6 indicating active disease.^33^

#### Medication adherence

Participants used a visual analog scale to rate their past‐month adherence to all reported medications, specific and nonspecific to cSLE, from 0% to 100%, with scores ≥80% indicating adequate adherence.^34^ This approach, adapted from the Medication Adherence Self‐Report Inventory,^35^ is similar to prior studies of youth with cSLE.^36^ We report average adherence across all medications reported for each participant.

#### HRQoL

Adolescent participants and their caregiver completed the Pediatric Quality of Life Inventory (PedsQL)^37^ and PedsQL Rheumatology Module.^38^ Items are scored on a five‐point Likert scale (0 = never, to 100 = almost always), with higher scores suggesting better quality of life.

#### Adverse events

TEACH recipients completed the 20‐item Negative Effects Questionnaire to assess potentially adverse events experienced during the intervention (eg, “I had more trouble sleeping”) and whether they were associated with TEACH (yes/no). For each endorsed event, participants also reported how negatively they were affected (0 = not at all, to 5 = extremely). Adverse events were monitored throughout the study.

### Covariate measures

#### Demographics and disease manifestations

Participant's age, sex assigned at birth, race, ethnicity, disease duration (months), and caregiver's highest level of education were reported by caregivers for adolescents and self‐reported by young adults at baseline. Rheumatologists reported participants’ disease manifestations at baseline using the Systemic Lupus International Collaborating Clinics classification criteria for SLE.^39^

#### Adverse childhood experiences

Exposure to nine adverse childhood experiences (ACEs; eg, violence, discrimination) was reported by adolescents’ caregivers and self‐reported by young adults, using items based on the National Survey of Children's Health ACEs measure.^40^ Endorsed exposures were scored as 1, with summed scores ranging from 0 to 9.^40^ Youths’ summed exposures were included as a covariate given known associations with pediatric mental health outcomes, pain, and fatigue.^41^

#### Medication

Participants’ medication usage (eg, name, indication) was collected from caregivers or self‐reported by young adults at baseline.

### Statistical analyses

#### Primary outcomes

TEACH feasibility and acceptability were examined using recruitment and retention rates, as well as qualitative interviews. Recruitment/retention rates and descriptive statistics were calculated using IMB SPSS Statistics (version 20). Recorded and transcribed participant interviews were reviewed by three trained coders (a licensed psychologist, psychology doctoral student, and postbaccalaureate research coordinator) for quotes supporting a priori and emerging themes until saturation and consensus were achieved.^42^ This approach is similar to our prior work^19^ and based on grounded theory and thematic analytic frameworks.^43^, ^44^

#### Secondary and exploratory outcomes

A priori power analysis for a repeated measures analysis of variance with an α of 0.05 determined that a sample size of 60 would give 76% power to detect a medium to large effect (effect size = 0.6) on fatigue.^45^ Analyses for secondary and exploratory outcomes were conducted using R Statistical Software (version 4.4.2) on an intent‐to‐treat basis, and missing data were handled using imputation by chained equations with predictive mean matching. This approach was used to create 55 complete imputed data sets, accounting for total sample missingness.^46^ The imputation model included the focal predictors and covariates used in the analysis of covariance (ANCOVA) modeling described below, a binary indicator of postassessment attendance, longitudinal relationships between baseline and postassessment measures of each outcome, and cross‐sectional relationships among outcomes measured at the same time. Parameter estimates were pooled across analyses run on each imputed data set.

Separate ANCOVA models examined the effect of TEACH on secondary and exploratory outcomes at postassessment. Each model included treatment arm (TEACH and TAU or TAU alone), country and age strata, mean‐centered baseline measurements of the outcome, and relevant covariates (participant's race, ethnicity, and ACE exposures), which may have important effects on the outcomes above and beyond treatment exposure. Interactions of treatment arm with the baseline measurement of the outcome were added to determine whether TEACH effects varied based on participants’ baseline symptom levels. Interaction terms were retained when model fit was improved and otherwise discarded. Notably, HRQoL models were estimated for adolescent participants only because these measures were normed for, and completed by, youth <18 years of age and their caregivers.

Main effects are quantified by the difference in estimated marginal means (emms) between treatment arms. Specifically, we used the R emmeans package to compare average predicted responses across treatment arms while adjusting for other covariates. Interaction effects are described by adjusted beta (adj β) coefficients and plotted to facilitate interpretation (see Supplementary Figures 2 and 3). All reported confidence intervals (CIs) used a 95% confidence level. Data will be shared upon reasonable request.

## RESULTS

A total of 200 youth were approached from August 2020 to March 2023, 97 of whom (48.5%) consented to participate. Of these, 10 were lost to follow‐up, 2 did not meet inclusion criteria, and 21 were ineligible based on screening results (see Figure 1). Thus, the analytic sample included 64 youth with cSLE (mean age [±SD] 16.5 [±2.1] years, age range 12 to 20 years; 62 were female [96.9%]). The majority were recruited from the United States (39 [60.9%]) versus Canada and were adolescents (<18 years old; 41 [64.1%]). The most commonly reported medications at baseline included hydroxychloroquine (*n*_*TEACH and TAU*_ = 21), *n*_*TAU*_ = 25), hycophenolate mofetil (*n*_*TEACH and TAU*_ = 15, *n*_TAU_ = 14), and prednisone (*n*_*TEACH and TAU*_ = 12, *n*_*TAU*_ = 7).

A total of 21 youth (33%) qualified on one screening measure, 22 (34%) on two screening measures, and 21 (33%) on all three screening measures. At baseline, 40 (63%) enrolled participants showed elevated fatigue symptoms, 44 (69%) showed elevated pain symptoms, and 44 (69%) showed elevated depressive symptoms. The two arms are described in Tables 1 and 2. At the eight‐week postassessment, 14 participants evidenced clinically significant reductions in raw (vs model estimated) fatigue symptom scores (10 [31.3%] TEACH and TAU; 4 [12.5%] TAU alone), 25 in raw pain symptom scores (15 [46.9%] TEACH and TAU; 10 [31.1%] TAU alone), 6 in raw anxiety symptom scores (5 [15.6%] TEACH and TAU; 1 [3.1%] TAU alone), and 23 in raw depression symptom scores (19 [59.4%] TEACH and TAU; 4 [12.5%] TAU alone). A total of 38 adverse events (eg, “I feel more worried”) were reported by TEACH and TAU completers, though only 19 were related to TEACH and none deemed potentially harmful. The percentage of missing data ranged from 0% to 17%, primarily occurring at postassessment due to participant dropout. There was no evidence of selective attrition, and data were deemed missing at random.

**Table 1:** Baseline demographic characteristics of the participants (n = 64)*

Characteristic	Randomized participants, No. (%)	
	TEACH and TAU (n = 32)	TAU alone (n = 32)
Age, mean (SD)	16.6 (±2.2)	16.6 (±2.0)
Sex, n (%)		
Female	31 (96.9)	31 (96.9)
Male	1 (3.1)	1 (3.1)
Race, n (%)^a^		
Asian	8 (25.0)	8 (25.8)
Black	7 (21.9)	7 (21.9)
Other^b^	5 (15.6)	1 (3.2)
White	12 (37.5)	15 (48.9)
Missing	N/A	1 (3.1)
Ethnicity		
Hispanic	10 (31.3)	4 (12.9)
Non‐Hispanic	22 (68.8)	27 (87.1)
Missing	N/A	1 (3.1)
Country		
United States	20 (62.5)	19 (59.4)
Canada	12 (37.5)	13 (40.6)
Caregiver education^c^		
Less than high school	1 (3.1)	4 (12.5)
High school degree or equivalent	8 (25.0)	8 (25.0)
Partial college/trade school	2 (6.3)	1 (3.1)
Trade certificate, diploma, or associate degree equivalent	3 (9.4)	1 (3.1)
College degree or greater	13 (40.6)	12 (37.5)
Missing	5 (15.6)	6 (18.8)
≥1 ACEs (0–9)	14 (43.8)	12 (37.5)
Missing	4 (12.5)	4 (12.5)

* ACE, adverse childhood experience; N/A, not applicable; TAU, treatment as usual; TEACH, Treatment and Education Approach for Childhood‐Onset Lupus.

^a^ Race and ethnicity are social constructs used to characterize the representativeness of the sample and generalizability of the results relative to the population with childhood‐onset systemic lupus erythematosus.

^b^ Other includes Native Hawaiian, Other Pacific Islander, and American Indian; these were consolidated owing to small numbers.

^c^ Caregiver education was the highest level of education reported by the adolescents’ primary caregiver.

**Table 2:** Baseline health characteristics of the participants (n = 64)*

Characteristic	Randomized participants	
	TEACH and TAU (n = 32), mean (±SD)	TAU alone (n = 32), mean (±SD)
Youth symptoms		
Depressive symptoms, T‐score	63.6 (±12.0)	64.4 (±10.4)
Fatigue, T‐score	61.6 (±8.5)	60.1 (±6.7)
Pain intensity (0–10)	3.5 (±2.0)	4.0 (±1.9)
Anxiety symptoms, Z‐score	0.1 (±1.1)	−0.1 (±0.8)
cSLE‐related factors		
Time since diagnosis, median (range)	31 (1–192)	26 (0–178)
Missing	N/A	1 (3.1)
Medications, median (range)	2 (0–12)	2 (0–10)
Missing	1 (3.1)	2 (6.3)
Medication adherence (0–100)	83.7 (±15.7)	84.1 (±19.2)
Missing	2 (±6.3)	N/A
Disease manifestations (0–38)	0.50 (±0.75)	0.53 (±1.0)
Missing	4 (±12.5)	2 (±6.3)
Disease activity (0–105), median (range)	5 (0–18)	2 (0–12)
Missing	4 (12.5)	3 (9.4)
HRQoL^a^ general adol (0–100)	54.9 (±18.2)	63.6 (±17.7)
Missing	12 (±37.5)	12 (±37.5)
HRQoL rheumatology adol (0–100)	64.8 (±17.3)	68.6 (±18.0)
Missing	12 (±37.5)	12 (±37.5)
HRQoL general adult (0–100)	51.7 (±13.8)	61.0 (±15.3)
Missing	12 (±37.5)	12 (±37.5)
HRQoL rheumatology adult (0–100)	63.7 (±13.4)	66.4 (±16.0)
Missing	12 (±37.5)	12 (±37.5)

* Depressive symptoms were measured using the Children's Depression Inventory 2nd Edition for participants younger than 18 years old and the Beck Depression Inventory II for participants older than 18 years old. Fatigue was measured using the PROMIS Pediatric Fatigue Short Form for participants younger than 18 years old and the Adult PROMIS Fatigue Short Form for participants older than 18 years old. Anxiety symptoms were measured using the Screen for Child Anxiety Related Disorders for participants younger than 18 years old and the Screen for Adult Anxiety Related Disorders for participants older than 18 years old. Medication adherence was measured using a visual analog scale based on the Medication Adherence Self‐Report Inventory. Disease manifestations were measured using the Systemic Lupus International Collaborating Clinics classification. Disease severity was measured with the Systemic Lupus Erythematosus Disease Activity Index. adol HRQoL was measured using PedsQL and PedsQL Rheumatology Module completed by both the adol and their caregiver. adol, adolescent; cSLE, childhood‐onset systemic lupus erythematosus; HRQoL, health‐related quality of life; PedsQL, Pediatric Quality of Life Inventory; PROMIS, Patient‐Reported Outcomes Measurement Information System; TAU, treatment as usual; TEACH, Treatment and Education Approach for Childhood‐Onset Lupus.

^a^ HRQoL measures were completed by adol patients (<18 years old) and their caregivers (“adult”).

### Primary outcomes

Of the 200 identified eligible youth, 97 (48.5%) consented to participate in the study (recruitment), whereas 59 (92.2%) of the 64 participants randomized into one of the treatment arms completed the eight‐week postassessment (retention; Table 3). The most common reasons for declining participation were lack of interest or time. An unconditional, exact test on the difference in proportions^47^ (Δ_p_ −0.03, 95% CI −0.28 to 0.20) failed to detect a difference in retention rates across treatment arms (*P* = 0.75). Average fidelity across intervention sessions was 98%.

**Table 3:** Recruitment and retention rates*

Location	Approached, n	Consented, n (% of approached)	Enrolled, n	Completed study, n (% of enrolled)	Randomized to TEACH, n	Completed TEACH, n (% randomized to TEACH)
United States	106	58 (54.7)	39	36 (92.3)	20	17 (85.0)
HDVCH	36	20 (55.6)	12	11 (91.7)	–	–
Seattle	29	18 (62.1)	15	13 (86.7)	–	–
Montefiore	25	13 (52.0)	9	9 (100.0)	–	–
CCHMC	11	5 (45.5)	2	2 (100.0)	–	–
UAB	5	2 (40.0)	1	1 (100)	–	–
Canada	94	39 (41.5)	25	23 (92.0)	12	12 (100.0)
Total	200	97 (48.5)	64	59 (92.2)	32	29 (90.6)

* CCHMC, Cincinnati Children's Hospital Medical Center; HDVCH, Helen DeVos Children's Hospital; TEACH, Treatment and Education Approach for Childhood‐Onset Lupus; UAB, University of Alabama.

Interviews were completed by 34 youth, 22 of whom received TEACH initially and 12 of whom received TEACH clinically after the postassessment. Four themes were identified that supported TEACH acceptability: (1) the logical connection among TEACH skills, (2) ease of use, (3) an immediate effect of specific skills, and (4) factors supporting a positive interventionist/participant relationship. Illustrative participant quotes are reported in Table 4. Regarding the relationship among TEACH skills, interviewees reported that the skills were logically connected and went “hand in hand.” Although some desired additional sessions, they also reported ease of use and positive impacts. Namely, participants referenced frequent use of relaxation skills including mindful breathing and progressive muscle relaxation, which they found helpful and accessible. Participants also noted using pleasant activity planning and/or activity pacing to prevent symptom exacerbations. The participant/interventionist relationship was cited as an important motivation for practicing skills, and no participants found that interventionist characteristics (eg, race, sex) impacted their perception of care. Although participants struggled to remember the names of TEACH skills, they reported practicing regardless. Greater symptom severity and busy schedules were identified as barriers to using TEACH skills, though participants enjoyed the virtual format and noted that remote delivery facilitated attendance.

**Table 4:** Participant quotations*

Theme	Participant quotations
Subdomain 1: practicability and usability of skills	
Relationships among skills	“A lot of strategies worked hand in hand … that's why they were so easy to incorporate because you can use multiple strategies at the same time …” “It was nice to be able to build on each skill from one week to the next.”
Ease of use	“It felt like something I can do on throughout my life.” “You can do it [practice skills] anywhere, anytime, and they do not take a lot of time as well so…”
Immediate effect of skills	“I had so much on my mind… but after I practiced it [breathing]… I had less overwhelming stress.” “I often struggled with controlling my breathing and mindfulness … when I was overwhelmed … I panicked … I used the tools taught in the program in my daily life outside of our meetings.”
Interventionist–participant relationship	“Even though it was like over the screen. we were still like able to make a bond and stuff like that, like if we were in‐person.” “I knew her very well after like one or two sessions because she treated me not like a patient but more like a friend.”
Subdomain 2: treatment outcomes	
Better sleep and self‐management	“I had a nightmare one night, and I couldn't take my mind out of it … my body was numb … [after using progressive muscle relaxation] I was able to be present with myself and fall back asleep.” “Lupus … messes with your moods. It's not gonna ever be a day where I'm not feeling well because of the medications I have, so the methods I have that I've been taught … will be helpful.” “Well, it helped with like, the pain, like activity pacing helped with break stuff, so like that way, so like it wouldn't hurt, I guess. So, like taking breaks throughout doing something so at the end it wouldn't hurt.”
Lupus mind–body connection	“…[the] human mind is really powerful, and we realize that sometimes some things like I could prevent, or I could help better with simple things of enjoying doing the things I enjoy or taking a break from things that are making me experience those symptoms so yeah I think that's good.” “…they focused a lot about how that stress can impact your physical symptoms too. So now it's like, you know that you have those breathing techniques and the ways to calm down…which then won't…have as many physical symptoms.”
Subdomain 3: detractors from practice and use of skills	
Forgotten skills	“Sometimes they're hard to remember because … [you're learning] a lot of new skills …”
Severity of symptoms	“The phase when you're out of remission might be a phase that you're in a very dark place, physically and mentally in terms of lupus, so. Like you might think that these activities might not be helpful because of the intense situation you're in.”
Busy schedule	“I work, I'm diving (I'm there all the time), and then just like hanging out with my friends, too. Because I do that a lot. And it's just like taking up my day and I just don't have any time for… just to… put that into my schedule.” “…busy with school and all that, you kind of forget sometimes.”
Subdomain 4: program format	
TEACH format	“I feel like I was able to let loose more because it was video because I was able to put off my camera and just speak my mind and be like this person doesn't know me at the end of the day…” “I wouldn't mind if it was in person either … but I feel like virtual is so easy for everybody to do just because … people have their own lives to do and it's easier to navigate through their lives …”
Too compact	“I feel like it ended a little bit too quick.”

* TEACH, Treatment and Education Approach for Childhood‐Onset Lupus.

### Secondary and exploratory outcomes

ANCOVA modeling revealed effects of TEACH on depressive and fatigue symptoms but not pain intensity (see Table 5). Specifically, TEACH participation was associated with a 7.88‐point (14%) decrease in depressive symptom T‐scores (*C*_emm_ 7.88, 95% CI 3.20–12.60) relative to TAU alone, adjusting for covariates (eg, race, ethnicity, country and age strata, ACE exposure). TEACH participation was also associated with a 3.91‐point (7%) average reduction in fatigue T‐score from baseline (*C*_emm_ 3.91, 95% CI 0.44–7.39). There was an interaction of treatment arm with baseline fatigue (adj β 0.55, 95% CI 0.04–1.05; Supplementary Table 2), such that TEACH exerted a larger effect on postassessment fatigue for participants with lower levels of baseline fatigue (see Supplementary Figure 2). There were no significant effects of TEACH on average pain intensity.

**Table 5:** Difference in postassessment estimated marginal means for treatment groups (n = 64)*

Characteristic	Estimated marginal mean (95% CI)		Difference in marginal means (95% CI)	*P* value
	TAU alone	TEACH and TAU		
Secondary outcomes				
Depressive symptoms, T‐score	58.0 (53.3 to 62.7)	50.1 (46.4 to 53.8)	7.88 (3.20 to 12.60)	0.001
Fatigue, T‐score	55.8 (52.3 to 59.3)	51.9 (49.1 to 54.7)	3.91 (0.44 to 7.39])	0.028
Pain intensity (0–10)	3.4 (2.5 to 4.3)	2.5 (1.7 to 3.2)	0.89 (−0.06 to 1.84)	0.06
Exploratory outcomes				
Anxiety symptoms, Z‐score	0.05 (−0.30 to 0.40)	−0.30 (−0.57 to −0.02)	0.35 (0.01 to 0.69)	0.04
Disease activity (0–105)	5.6 (2.8 to 8.4)	5.1 (2.5 to 7.7)	0.49 (−2.08 to 3.06)	0.695
Medication adherence (0–100)	92.3 (85.4 to 99.1)	93.1 (87.4 to 98.7)	−0.80 (−7.67 to 6.06)	0.813
HRQoL^a^ general child (0–100)	66.5 (57.1 to 75.8)	64.4 (56.0 to 72.9)	2.06 (−6.53 to 10.70)	0.618
HRQoL rheumatology child (0–100)	65.7 (56.3 to 75.0)	68.8 (61.1 to 76.5)	−3.13 (−13.2 to 6.90)	0.522
HRQoL general adult (0–100)	62.1 (55.3 to 68.9)	62.0 (55.7 to 68.3)	0.10 (−7.36 to 7.56)	0.978
HRQoL rheumatology adult (0–100)	70.7 (63.5 to 77.9)	71.9 (65.4 to 78.5)	−1.24 (−8.54 to 6.07)	0.730

* Results for each outcome are averaged over the levels of country, age, race, ethnicity, and the corresponding pretest value for each covariate. Data are differences in estimated marginal means, a predicted value for the outcome averaged across levels of other categorical covariates in the model. Differences and 95% CIs are in the metric of each postassessment outcome. CI, confidence interval; HRQoL, health‐related quality of life; TAU, treatment as usual; TEACH, Treatment and Education Approach for Childhood‐Onset Lupus.

^a^ HRQoL measures were completed by adolescents (≤18 years old; “child”) and their caregivers (“adult”).

Results from exploratory analyses suggest effects of TEACH on anxiety symptom Z‐scores (*C*_emm_ 0.35, 95% CI 0.01–0.69; Table 5). Although there was no significant difference in marginal mean medication adherence scores between treatment arms (*C*_emm_ −0.80, 95% CI −7.67 to 6.06), there was an interaction effect of treatment arm with baseline medication adherence (adj β −0.49, 95% CI −0.88 to −0.10). Namely, Supplementary Figure 3 illustrates that TEACH was associated with improved postassessment medication adherence for participants with poor baseline adherence. There was no effect of TEACH on disease activity or HRQoL.

## DISCUSSION

TEACH, a brief, individually and remotely delivered cognitive behavioral intervention, is feasible and may improve depressive, fatigue, and anxiety symptoms in youth with cSLE compared to medical TAU alone. Thus, common and debilitating symptoms impacting people with cSLE are potentially modifiable with nonpharmacologic intervention. Despite the great need, there is extremely limited research on psychosocial interventions for youth with cSLE,^13^, ^14^ and this is the first trial to show a positive impact on patient outcomes. These findings highlight the potential importance of augmenting medical care with behavioral strategies to optimize patient outcomes, echoing recent calls to increase recognition, identification, and prompt management of mental health and associated symptoms in pediatric rheumatology populations.^48^

Our primary goal was to evaluate TEACH feasibility. Promisingly, our retention rate and qualitative data speak to the value of the intervention and suggest that we were able to build trusting and impactful relationships after engaging youth into the study. Though our hypothesized recruitment rate was >65%, this estimation was made from behavioral trials with other pediatric chronic pain populations (recruitment rates of >75%).^49^ Our reported recruitment rate is similar to that of our prior treatment study of youth with cSLE,^14^ which may suggest systematic barriers to care for this population. Further work is needed to identify and address these barriers, but sociocultural factors (eg, marginalization, systemic racism), lasting impacts of the COVID‐19 pandemic, and mental health stigma may play important roles. Emerging work in other pediatric conditions disproportionately impacting Black youth (eg, sickle cell disease)^16^ have achieved excellent recruitment for intervention studies by engaging people with lived experience to collaboratively refine and adapt approaches to care. We took a similar approach developing the TEACH protocol^16^ but may consider how to augment the role of patient coinvestigators moving forward. Notably, among the 75 youth who declined participation, many cited lack of interest or time. To address these challenges, we will continue to partner with our clinical partners and patient coinvestigators to develop engaging recruitment tools (eg, videotaped participant testimonials) as well as flexible approaches to intervention delivery that may better fit youths’ schedules.

TEACH completers found the program beneficial, the live virtual format convenient, and the relationship with their interventionist valuable. Although research suggests that people from racially minoritized groups may prefer to work with those of similar backgrounds,^50^ our qualitative findings suggest that patient–interventionist racial and/or sex discordance did not impact the high levels of trust and support fostered with interventionists. Interestingly, some participants requested additional sessions and others forgot the names of skills learned. Therefore, booster sessions may be important, particularly given the neuropsychiatric symptoms associated with cSLE and potential implications for supporting memory/cognitive functioning.

Findings suggest effects of TEACH on participants’ depressive, fatigue, and anxiety symptoms. Continued work with additional time points will be important to determine if these effects, like those of psychological interventions for pediatric chronic pain conditions,^17^ persist beyond initial follow‐up. Examination of more distal outcomes may inform future adaptations of the intervention, such as the inclusion of booster sessions. Notably, although findings suggest an impact of TEACH on depressive symptoms, relatively few youth evidenced a clinically significant improvement in their raw symptom scores. However, the threshold for determining clinical significance is steep (eg, ≥50% reduction), particularly when clinical depression was not a universal inclusion criterion. Thus, more moderate symptom reductions are still meaningful and may suggest the potential for TEACH to reduce the risk of symptom persistence or progression.

We also identified differential effects of TEACH on fatigue depending on baseline symptoms, such that participants with less baseline fatigue derived greater benefits than those with greater fatigue. Fatigue may be a proxy for disease severity and/or may impact youths’ ability to learn intervention skills. These findings suggest the importance of early intervention (ie, offering support as soon as is feasible after diagnosis) in line with recent guidance for supporting mental health concerns in pediatric rheumatology.^48^ Importantly, anxiety as well as depressive symptoms improved following TEACH. This is noteworthy because anxiety symptoms were not a requirement for study enrollment but were common and improved. There was a trend toward improved pain, but differences did not reach statistical significance, possibly due to relatively low levels of baseline pain.^3^ Although there was no overall change in average medication adherence across groups, exploratory analyses suggest that TEACH was associated with improved medication adherence among those with poor baseline adherence. This observation underscores the importance of nonpharmacologic approaches to support medication adherence, which directly impacts disease control and other clinical outcomes in SLE.

Limitations to this study include our relatively small sample size, which may have negatively impacted statistical power and the generalizability of these results. However, ours is the largest trial of an effective cognitive behavioral intervention for youth with cSLE. Relatedly, our eligibility criteria and the length of the program may have resulted in selection bias away from youth with more severe mental health problems and/or disease activity. However, average levels of disease activity, pain intensity, and mental health symptoms in this sample are comparable to those reported for other cohorts with cSLE, allaying these concerns to some degree.^3^ Notably, youth could qualify for participation in this study based on only one symptom (eg, depressive, pain, or fatigue) versus all three in an effort to make TEACH broadly inclusive of a diverse patient population and to reach patients who required more than universal screening and education,^48^ but this may have not met severity thresholds for referral to additional specialties. Subsequent work may include youth with more severe symptoms, the inclusion of whom may increase the magnitude of TEACH treatment effects given the greater potential room for improvement (as per our findings for medication adherence). Third, our decision to exclude youth receiving psychological treatment for depressive, fatigue, or pain symptoms preserved design integrity but may have further limited generalizability. Similarly, given the multisite nature of the study, features of the TAU condition may vary among sites. Future work may include more detailed assessments of both TAU and other adjunctive therapies used by youth and families. Finally, our measure of medication adherence was reported by patients and therefore vulnerable to response bias. Given the potential positive effects of TEACH participation on medication adherence, future investigation using more comprehensive assessment approaches is warranted.

There is a clear need for multidisciplinary care in cSLE and a growing recognition of the importance of mental health and associated symptoms to youths’ overall well‐being.^48^ Our findings support the feasibility and acceptability of an innovative, nonpharmacological intervention for youth with cSLE that leverages the trust and accessibility of the rheumatologic setting to overcome potential barriers and engage youth into behavioral care. Ours is the first trial to demonstrate a measurable impact of a brief, tailored CBT program on some of the most common and debilitating symptoms faced by patients with cSLE. Therefore, this study may have significant implications for the care of youth with cSLE and future work seeking to expand access to effective, nonpharmacological intervention.

## AUTHOR CONTRIBUTIONS

All authors contributed to at least one of the following manuscript preparation roles: conceptualization AND/OR methodology, software, investigation, formal analysis, data curation, visualization, and validation AND drafting or reviewing/editing the final draft. As corresponding author, Dr Cunningham confirms that all authors have provided the final approval of the version to be published and takes responsibility for the affirmations regarding article submission (eg, not under consideration by another journal), the integrity of the data presented, and the statements regarding compliance with institutional review board/Declaration of Helsinki requirements.

## Supporting information


**Disclosure Form**:

**Supplemental Table 1** Qualitative Interview Guide
**Supplemental Table 2**. Effect of TEACH on secondary and exploratory outcomes (n=64)
**Supplemental Figure 1**. TEACH Content
**Supplemental Figure 2**. Interaction Effect of Treatment Arm and Baseline (Pretest) Fatigue on Posttest Fatigue
**Supplemental Figure 3**. Interaction Effect of Treatment Arm and Baseline (Pretest) Medication Adherence on Posttest Medication Adherence
