# Supplementary material for: Cognitive Behavioral Therapy for Youth With Childhood‐Onset Lupus: A Randomized Clinical Trial
Source: Arthritis Care Res (Hoboken). 2026 Mar 20;78(8):1024–35. doi: 10.1002/acr.70010 (PMC13420951; doi:10.1002/acr.70010)
Supplement: Supplementary file 2 — Supplemental Table 1 Qualitative Interview Guide Supplemental Table 2. Effect of TEACH on secondary and exploratory outcomes (n=64) Supplemental Figure 1. TEACH Content Supplemental Figure 2. Interaction Effect of Treatment Arm and Baseline (Pretest) Fatigue on Posttest Fatigue Supplemental Figure 3. Interaction Effect of Treatment Arm and Baseline (Pretest) Medication Adherence on Posttest Medication Adherence [file ACR-78-1024-s002.docx]

Supplemental Table 1. Qualitative Interview Guide

Supplemental Table 2. Effect of TEACH on secondary and exploratory outcomes (n=64)

Supplemental Figure 1. TEACH Content

Supplemental Figure 2. Interaction Effect of Treatment Arm and Baseline (Pretest) Fatigue on Posttest Fatigue

Supplemental Figure 3. Interaction Effect of Treatment Arm and Baseline (Pretest) Medication Adherence on Posttest Medication Adherence

**Supplemental Table 1.** Qualitative Interview Guide

| **Theme** : Feasibility |  |  |
| --- | --- | --- |
| **Initial Query^a^:** | **Follow up:** |  |
| Do you think this remotely delivered program was a good way to help you cope with your lupus symptoms?  What symptoms specifically did the TEACH program help you with? |  |  |
| Do you think this program would be helpful to others with lupus? |  |  |
| Would you use any of the skills at home to cope  with your lupus symptoms? | If so, which skills do you think you would use? |  |
| Do you think you will be able to integrate the skills taught in this program into your schedule with school/work and any other activities you are involved in? |  |  |
| What might get in the way of you practicing these skills on your own? |  |  |
| **Theme:** Tolerability & Acceptabilit**y** |  |  |
| **Initial Query:** | **Follow up:** |  |
| How is the pace of the program?  How did you feel about remote intervention  delivery?  Did you like the way that coping skills like progressive muscle relaxation and diaphragmatic breathing, were explained for the sessions?  For the parts of the sessions that focused on sleep,  did you like the way the skills were taught?    For the parts of the sessions that focused on mindfulness, did you like the way the skills were taught?  For the parts of the sessions that focused thoughts related to your lupus (using calming statements and understanding how thoughts and beliefs impact physical symptoms), did you like  the way the skills were taught?  For the parts of the sessions that focused on increasing pleasant activities and activity pacing, did you like the way the skills were taught?  For the parts of the sessions that focused on problem solving, did you like the way the skills were taught?  For the parts of the sessions that focused on advocating for yourself, did you like the way skills were taught?  Did your interventionist specifically address communication skills with you (note this is optional content)?  Over 18: For the parts of the sessions that focused  on medication adherence, did you like the way the  skills were taught? | Did we start with skills that felt easier and then move on to more advanced skills at a good speed?  Did it seem like an appropriate way to learn the content?  Were there strategies during the sessions that felt too hard? If yes, which ones?  Did the program move too quickly? Explain.  Did the program move too slowly? Explain.  If yes: what did you like about it?  If no: what didn’t you like?  How could the skills be presented better?  In general, how well do you feel you could use the skills in the future?  If yes: what did you like about it?  If no: what didn’t you like?  How could the skills be presented better?  In general, how well do you feel you could use the skills in the future?  If yes: what did you like about it?  If no: what didn’t you like?  How could the skills be presented better?  In general, how well do you feel you can use the skills in the future?  If yes: what did you like about it?  If no: what didn’t you like?  How could the skills be presented better?  In general, how well do you feel you could use the skills in the future?  If yes: what did you like about it?  If no: what didn’t you like?  How could the skills be presented better?  In general, how well do you feel you can use the skills in the future?  If yes: what did you like about it?  If no: what didn’t you like?  How could the skills be presented better?  In general, how well do you feel you can use the skills in the future?  If yes: what did you like about it?  If no: what didn’t you like?  How could the skills be presented better?  In general, how well do you feel you can use the skills in the future?  If yes: did you like the way the skills were taught?  What did you like about it?  If no: what didn’t you like?  How could the skills be presented better?  In general, how well do you feel you can use the skills in the future?  If yes: what did you like about it?  If no: what didn’t you like?  How could the skills be presented better?  In general, how well do you feel you can use the skills in the future? |  |
| **Theme:** Content |  |  |
| **Initial Query** | **Follow up** |  |
| Did the coping skills hold your interest? | What were some of your favorites? Why?  Which ones did you like less? Why?  Were there certain skills that you liked more than the others? If so, why? |  |
| **Theme:** Format |  |  |
| **Initial Query** | **Follow up** |  |
| How do you feel about the format of the program?  How did you feel about the idea of 6 sessions?  Do you think there are enough sessions to adequately teach you about coping with your lupus?  At this time, we do not offer additional sessions, but would you participate in more sessions if they were available?  What are some of the benefits of this program? Are there any drawbacks?  Did you like that the program was delivered remotely?  How did you feel about scheduling the remote sessions? | Was it easier or harder than scheduling in person sessions? |  |
| Did you have any issues with accessing the remote intervention?  Do you feel that you could have the same connection with the interventionist over video chat as compared to seeing someone in-person? | Did you have any technology issues?  Did you have any issues with your surrounding during the sessions? (e.g., Was it too loud?) |  |
| Did you feel more, or less, comfortable completing the intervention remotely as compared to in-person?  Did you feel that the intervention was more/less private than an in-person session?  Did you feel that it was more/less easy to express yourself over video chat compared to in-person treatment? |  |  |
| **Other Questions** |  |  |
| **Initial Query** | **Follow up** |  |
| How confident are you that this program could help you cope with your lupus?  How often have you used skills to cope with your lupus on your own prior to learning about this program?  How likely do you think you would use coping skills on your own after completing such a program?  Did you experience any distress due to COVID-19? Did you feel that the TEACH program helped address any distress you felt from COVID-19?  Did you find anything motivating about the program?    Did you find anything discouraging about the program?  What did you think of the interventionist?  How helpful did you find the interventionist?  Did you feel that your experiences were adequately understood by the interventionist?  Did you feel that TEACH was individualized to you? (i.e., rather than feeling like your symptoms and experiences were like everyone else’s’.)  Have your thoughts about working with a psychological provider to manage your lupus changed since starting  this program? | *If a positive confidence is reported*: What part of this program seems most important in building that confidence?  *If less than positive confidence is reported:* What part of this program could be improved to better build your confidence?  If yes: What skills do you think help you the most? Did any other aspects of TEACH help with the distress you felt from COVID-19?  *If no:* Is there anything that would have been helpful? Is there anything that could have been better presented to address this?  Did you feel like you were able to make a connection?  Was there anything that would have helped you connect more to the interventionist?  Does the interventionist’s gender/age/race/other factors matter in building a relationship with the provider? How so?  Are you more open to working with a psychological provider now and in the future? |  |

**^a^**Interview was semi-structured. Initial query was always posed, and the appropriate follow up question followed.

**Supplemental Table 2:** Effect of TEACH on secondary and exploratory outcomes (n=64)^a^

| **Outcome/Parameter** | **Statistics** | | | | | **Model Fit Indices** | | |
| --- | --- | --- | --- | --- | --- | --- | --- | --- |
|  | **Adj. b** | **SE** | **95% CI** | **t-value** | ***p*** | **Adj. R^2^** | **95% CI** | **FMI** |
| **Secondary Outcomes** | | | | | | | | |
| Depressive Symptoms T-score |  |  |  |  |  | 0.47 | 0.28, 0.64 | 0.015 |
| TEACH | -7.88 | 2.33 | -12.55, -3.21 | -3.39 | 0.001 |  |  |  |
| Fatigue T-score |  |  |  |  |  | 0.35 | 0.16, 0.54 | 0.020 |
| TEACH | -3.91 | 1.73 | -7.38, -0.45 | -2.27 | 0.028 |  |  |  |
| Baseline Fatigue | 0.14 | 0.19 | -0.25, 0.52 | 0.71 | 0.480 |  |  |  |
| Arm x Baseline Fatigue | 0.55 | 0.25 | 0.04, 1.05 | 2.17 | 0.035 |  |  |  |
| Pain Intensity (0-10) |  |  |  |  |  | 0.39 | 0.20, 0.58 | 0.036 |
| TEACH | -0.89 | 0.47 | -1.83, 0.05 | -1.89 | 0.064 |  |  |  |
| **Exploratory Outcomes** | | | | | | | | |
| Anxiety Symptoms Z-score |  |  |  |  |  | 0.60 | 0.43, 0.74 | 0.027 |
| TEACH | -0.35 | 0.17 | -0.69, -0.01 | -2.06 | 0.045 |  |  |  |
| Disease activity (0-105) |  |  |  |  |  | 0.30 | 0.10, 0.52 | 0.271 |
| TEACH | -0.49 | 1.23 | -2.98, 2.00 | -0.40 | 0.693 |  |  |  |
| Medication Adherence (0-100) |  |  |  |  |  | 0.42 | 0.21, 0.61 | 0.180 |
| TEACH | 0.80 | 3.37 | -5.99, 7.60 | 0.24 | 0.813 |  |  |  |
| Baseline Adherence | 0.65 | 0.12 | 0.41, 0.89 | 5.53 | < 0.001 |  |  |  |
| Arm x Baseline Adherence | -0.49 | 0.20 | -0.88, -0.10 | -2.50 | 0.016 |  |  |  |
| HRQOL general-adol^b^ (0-100) |  |  |  |  |  | 0.64 | 0.35, 0.82 | 0.415 |
| TEACH | -2.06 | 4.05 | -10.42, 6.30 | -0.51 | 0.615 |  |  |  |
| HRQOL rheum-adol(0-100) |  |  |  |  |  | 0.46 | 0.17, 0.70 | 0.309 |
| TEACH | 3.13 | 4.81 | -6.77, 13.03 | 0.65 | 0.521 |  |  |  |
| HRQOL general-adult (0-100) |  |  |  |  |  | 0.57 | 0.27, 0.78 | 0.402 |
| TEACH | -0.10 | 3.60 | -7.49, 7.29 | -0.03 | 0.978 |  |  |  |
| HRQOL rheum-adult (0-100) |  |  |  |  |  | 0.48 | 0.21, 0.70 | 0.208 |
| TEACH | 1.24 | 3.53 | -6.00, 8.47 | 0.35 | 0.729 |  |  |  |

Abbreviations: TEACH, Treatment and Education Approach to Childhood-onset Lupus; Adj., adjusted; SE, standard error, CI, confidence interval, FMI, fraction of missing information; HRQOL, health-related quality of life; rheum, rheumatology

^a^Estimates derived from separate analysis of covariance (ANCOVA) models for each outcome. Each model adjusted for mean-centered baseline measurement, country and age strata, and participant demographics. Arm by baseline interaction terms were retained and shown only for models where they improved fit.

^b^HRQOL measures completed by adolescents (≤18 years; “adol”) and their caregivers (“adult”).

**Supplemental Figure 1.** TEACH Content

**
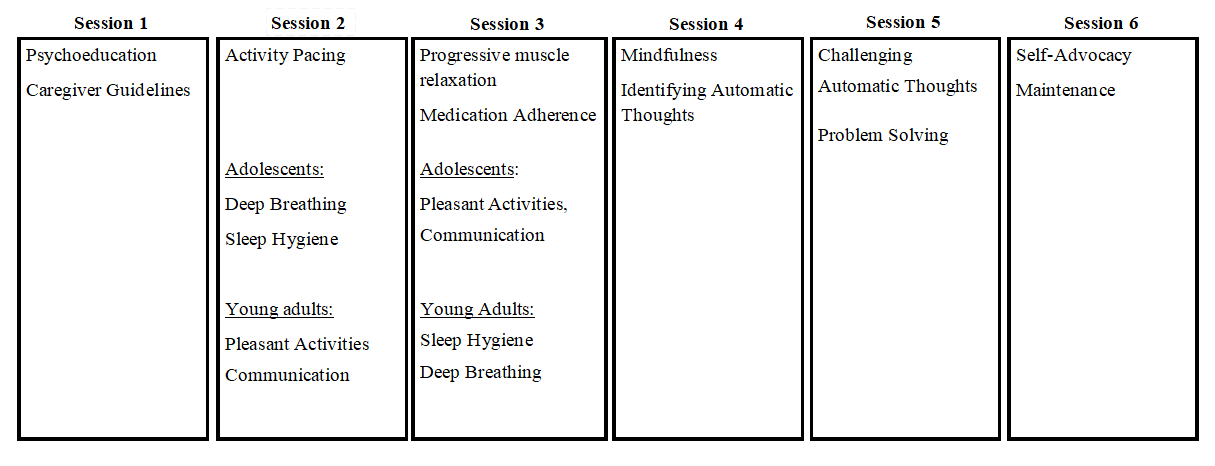
**TEACH session one and TEACH sessions four through six were delivered similarly for both age groups (adolescent and young adult). In TEACH session two and three, there was some variation in content presented to the two age groups.

**Supplemental Figure 2.** Interaction Effect of Treatment Arm and Baseline (Pretest) Fatigue on Posttest Fatigue


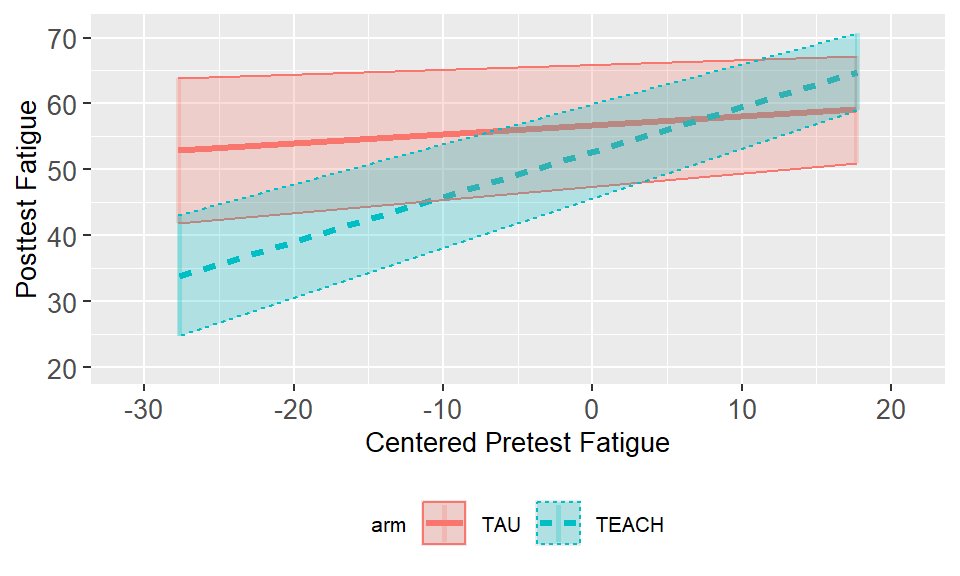


Pretest fatigue was mean centered, so 0 on the x-axis corresponds to the pretest mean (*M* = 60.8). The vertical distance between the lines is the intervention effect (difference in estimated posttest marginal means), which is not constant because of the interaction (i.e., the lines are not parallel). Low posttest fatigue is the best outcome. Patients with the lowest levels of pre-test fatigue (over 20 points below the mean) benefitted most from the TEACH intervention. The benefit disappeared for patients with pretest fatigue about 7 points above the mean.

Abbreviations: TAU, medical treatment as usual; TEACH, Treatment and Education Approach for Childhood Onset Lupus

**Supplemental Figure** **3.** Interaction Effect of Treatment Arm and Baseline (Pretest) Medication Adherence on Posttest Medication Adherence


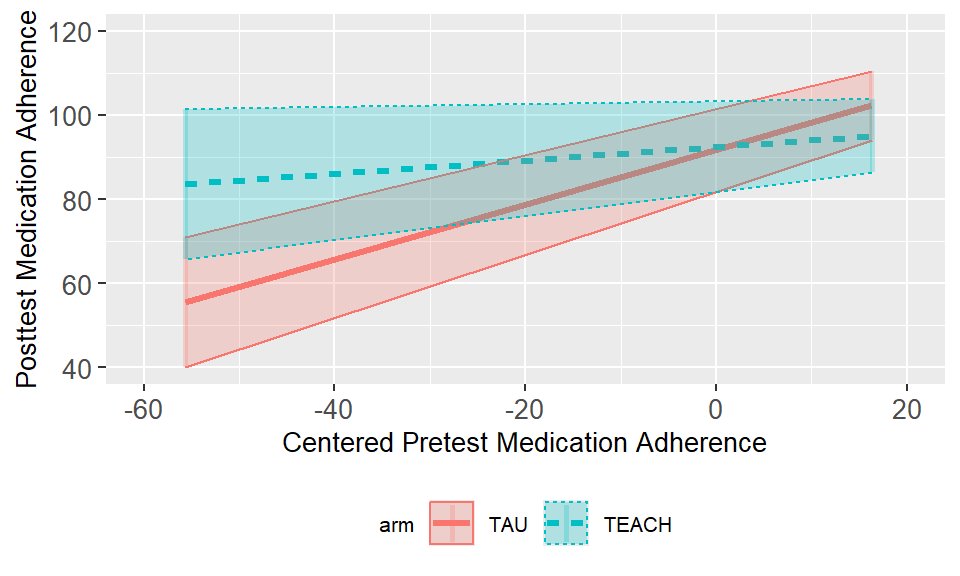


Pretest adherence was mean centered, so 0 on the x-axis corresponds to the pretest mean (*M* = 83.7). The vertical distance between the lines is the intervention effect (difference in estimated posttest marginal means), which is not constant because of the interaction (i.e., the lines are not parallel). High posttest adherence is the best outcome. Patients with the lowest levels of pre-test adherence (over 40 points below the mean) benefitted most from the TEACH intervention. The benefit disappeared for patients with pretest adherence very close to the mean.

Abbreviations: TAU, medical treatment as usual; TEACH, Treatment and Education Approach for Childhood Onset Lupus
